# Knowledge, attitudes, and practices regarding gout management among patients in Xiamen, China: a cross-sectional study

**DOI:** 10.3389/fpubh.2026.1786934

**Published:** 2026-06-17

**Authors:** Yonglong Yan, Yashuang Su, Wenhui Li, Qinfei Lin, Xin Du, Yu Li

**Affiliations:** 1 Department of Rheumatology, Xiang’an Hospital of Xiamen University, Xiamen, China; 2 Department of Rheumatology, Hebei General Hospital, Shijiazhuang, China; 3 Department of Physical Examination Center, Xiang'an Hospital of Xiamen University, Xiamen, China; 4 Shitang Community Health Service Center, Xiamen, China; 5 Department of Cardiac Surgery, Xiang’an Hospital of Xiamen University, Xiamen, China

**Keywords:** attitude, cross-sectional survey, gout, knowledge, patient education, practice

## Abstract

**Background:**

This study aimed to investigate the knowledge, attitude, and practice (KAP) of gout among patients with gout in the Xiamen region and identify factors influencing these domains to inform targeted interventions.

**Methods:**

A cross-sectional study was conducted in Xiamen, China. From November 2023 to January 2024, questionnaires were distributed via Questionnaire Star to collect demographic information and KAP scores. Structural Equation Modeling (SEM) was employed to explore the interrelationships among knowledge, attitudes, and practices.

**Results:**

A total of 622 questionnaires were collected, of which 462 were valid, with an effective rate of 74.3%. The median scores (IQR) were as follows: Knowledge, Media*n =* 16, IQR: 10–22; Attitude, Media*n =* 34, IQR: 29–37; and Practice, Media*n =* 40, IQR: 33–47. Significant positive correlations were found among the three domains: knowledge and attitude (r = 0.456, *p <* 0.001), knowledge and practice (r = 0.539, *p <* 0.001), and attitude and practice (r = 0.477, *p <* 0.001). Structural Equation Modeling demonstrated a good model fit (RMSEA = 0.068, SRMR = 0.069, TLI = 0.839, CFI = 0.851) and revealed that knowledge had a significant direct effect on practice (*β* = 0.644, *p <* 0.001), while the indirect effects through attitude were not statistically significant.

**Conclusion:**

Patients with gout demonstrated limited knowledge and moderately negative attitudes toward gout, yet relatively proactive self-management practices.

## Background

Gout is a prevalent chronic inflammatory disease that has shown a rising global burden, affecting approximately 1–4% of adults worldwide and imposing substantial public health and economic challenges. In China, recent epidemiological surveys report a prevalence ranging from 1.1 to 3.5%, with an increasing trend in coastal and urban regions. Gout typically progresses from hyperuricemia to advanced gout characterized by tophi and chronic arthritis (1–3). Dietary patterns such as high intake of seafood, red and processed meats, and refined grains contribute to elevated serum uric acid levels and increased gout risk (4). The warm and humid subtropical climate of Xiamen may further predispose residents to gout flares by influencing purine metabolism and dehydration-related uric acid concentration. Xiamen, a coastal Chinese city with a hot and humid climate, faces unique factors influencing gout’s prevalence and impact. China’s healthcare system operates through a hierarchical structure, with large hospitals serving as tertiary care centers and community health centers providing primary care services (5). There is an increasing trend toward managing chronic diseases like gout at the grassroots level.

A Knowledge, Attitude, and Practices (KAP) survey is a research instrument employed to assess a group’s understanding, attitudes, and behaviors regarding a specific subject, including diseases. This methodology serves as a diagnostic tool to gain insights into what is understood, believed, and put into practice in relation to various health conditions. In the context of health literacy, the KAP model is based on the premise that knowledge positively influences attitudes, subsequently shaping behaviors, which can have a direct impact on disease incidence and management (6–9). Previous KAP (Knowledge, Attitudes, and Practices) studies focusing on healthcare professionals have indicated that there may be a deficit in their understanding and implementation of best treatment practices for gout, potentially leading to inadequate patient education about the condition (10–12). Considering the increased incidence of gout in recent decades, coupled with an aging population, there is a growing concern that these factors may contribute to the escalating prevalence of gout (13). KAP surveys are valuable tools for assessing the knowledge, attitudes, and practices related to gout management in coastal urban populations. However, most previous studies have focused on healthcare providers rather than patients with gout (14), highlighting the need to investigate patient-level KAP patterns and influencing factors in regions such as Xiamen.

Therefore, this study aimed to investigate the KAP of gout among patients with gout in the Xiamen region, to provide a scientific basis for developing targeted health education and intervention strategies suited to the local dietary and environmental context.

## Methods

### Study design and subjects

This cross-sectional study was conducted from November 2023 to January 2024 among patients with gout at two medical institutions in Xiamen: Xiamen University Affiliated Xiang’an Hospital, and the Xiamen Haicang District Shitang Community Health Service Center. Ethical approval was obtained from the Medical Ethics Committee of Xiamen University, and informed consent was obtained from all participants. Participants were recruited using convenience sampling. Inclusion criteria for patients with gout encompassed individuals aged 18–80 years who had experienced at least one acute gout attack. Exclusion criteria involved the exclusion of questionnaires with completion times shorter than 2 s per single-choice question or 3 s per multiple-choice question, following established standards for online survey quality control (15).

### Questionnaire distribution

The survey was conducted from November 2023 to January 2024 at Xiamen University Affiliated Xiang’an Hospital and Xiamen Haicang District Shitang Community Health Service Center, which served as the main survey sites. Before distribution, investigators received standardized online training on questionnaire administration and data quality control. Participant identity and data authenticity were verified through WeChat account and IP address matching to ensure reliability. The questionnaire was designed and distributed via the Questionnaire Star online platform and completed by participants through WeChat scanning. To ensure data quality, the platform was configured so that each WeChat account was permitted only one submission, and each IP address was restricted to one submission; any duplicate submissions were automatically flagged and excluded. Questionnaires with abnormal completion times (less than 110 s or more than 1800 s) or logical errors were also excluded as invalid responses. All items were mandatory before data export and verification (15).

### Procedures

The design of the questionnaire drew upon insights and methodologies from previously published literature (16–18) and the “Merck Manual Gout^1^ “as valuable references content validity was assessed by a panel of three rheumatologists and two epidemiologists, who reviewed the questionnaire for relevance, clarity, and comprehensiveness. Subsequently, a pilot test was conducted on 53 gout patients with similar demographic characteristics to the target population (but excluded from the final study) to assess clarity and completion time. This resulted in a Cronbach’s *α* coefficient of 0.906, indicating a high level of internal consistency and reliability. The four-dimensional structure (demographics, knowledge, attitude, practice) reflects the standard KAP model framework, with a preceding demographic section to capture baseline variables.

The final questionnaire, conducted in Chinese, encompassed four distinct dimensions. Firstly, there was a section gathering Participant demographic information, comprising 14 questions. The Knowledge dimension followed, featuring 15 questions: seven single-choice questions (questions 1, 3–5, 13–15) were scored as 1 for correct responses and 0 for unclear or incorrect answers, while eight multiple-choice questions (questions 2, 6–12) carried a score range of 0–43. The Attitude dimension included 11 questions, all utilizing a five-point Likert scale, with scores ranging from 1 to 5, reflecting the degree of attitude, resulting in an overall score range of 11–55. Lastly, the Practice dimension consisted of 10 questions, also employing a five-point Likert scale, with scores ranging from 1 to 5, corresponding to the level of action taken, and yielding a total score range of 10–50.

### Sample size calculation

The sample size was calculated using the formula *n =* (z^2^p(1-p))/d^2^, where z = 1.96 at 5% level of significance and 5% acceptable margin of error (d = 0.05). The proportion of the expected population was set at 50%. Based on this calculation, the minimum required sample size was 384 (19).

### Statistical analyses

Statistical analysis was conducted using SPSS 26.0 (IBM Corp., Armonk, NY, USA). The Kolmogorov–Smirnov test confirmed that KAP scores were not normally distributed (*p <* 0.05); therefore, non-parametric tests were used. Comparisons between two groups were performed using the Mann–Whitney U test; for variables with three or more groups, the Kruskal-Wallis H test was used. Logistic regression was used to perform univariate and multivariate analysis. Due to the observed skewed distribution of scores, median values provided more robust measures of central tendency and more representative cut-off points than means. Dichotomizing scores at the median allowed us to use logistic regression to identify factors associated with scores in the upper half of the distribution. Pearson correlation analysis was employed to assess the correlations between knowledge, attitude, and practice scores. In multivariate analysis, median score was used as the cut-off value. Variables with *p <* 0.25 in univariate analysis were entered into multivariate logistic regression, as this more permissive threshold retains potentially explanatory variables that may be important in the adjusted model. Two-sided *p <* 0.05 were considered statistically significant in this study. Structural Equation Modeling (SEM) was used to explore the relationships between knowledge, attitudes, and practice, providing a framework for understanding these interrelationships and identifying key pathways. Statistical analysis was conducted using SPSS 26.0 (RRID:SCR_002865).

## Results

### Basic characteristics of participants

A total of 622 questionnaires were collected. Of these, 101 were excluded due to abnormal completion times (<110 s or more than 1800 s), and 59 were excluded because of logical errors (such as selecting both “correct” and “not sure” for the same question). Finally, 462 valid questionnaires were included, resulting in an effective response rate of 74.3%. Among the respondents, 251 (54.3%) were male, 337 (72.9%) had a university education or above, and 365 (79.0%) were employed full-time. The median scores (IQR) were as follows: Knowledge, Media*n =* 16, IQR: 10–22; Attitude, Media*n =* 34, IQR: 29–37; and Practice, Media*n =* 40, IQR: 33–47, indicating generally low knowledge, moderately negative attitudes, and relatively proactive practices toward gout management (Table 1).

**Table 1:** Baseline table.

Characteristics	*N* (%)	Knowledge (K)		Attitude (A)		Practice (P)	
		Median (IQR)	*P*	Median (IQR)	*P*	Median (IQR)	*P*
Total	462	16 (10, 22)		34 (29, 37)		40 (33, 47)	
Gender			0.884		0.924		0.716
Male	251 (54.3)	16 (9, 22)		33 (29, 37)		41 (32, 46)	
Female	211 (45.7)	16 (10, 22)		34 (29, 37)		40 (34, 48)	
Age (years)			0.041		<0.001		0.023
18–24	59 (12.8)	14 (5, 18)		29 (27, 34)		36 (30, 46)	
25 ~ 34	136 (29.4)	16 (11, 22)		34 (29, 37)		39.5 (32, 47)	
35 ~ 50	181 (39.2)	16 (9, 23.5)		34 (30, 37.5)		41 (36, 47.5)	
51 and above	86 (18.6)	16 (12, 21.3)		33 (31, 37)		41 (36.8, 46)	
Marital status			0.005		0.001		<0.001
Unmarried	109 (23.6)	14 (6, 18.5)		32 (27, 36)		37 (30, 46)	
Married	345 (74.7)	16 (11, 23)		34 (30, 37)		41 (36, 47)	
Divorced	5 (1.1)	13 (5.5, 13.5)		32 (26.5, 34.5)		38 (28.5, 42.5)	
Widowed	3 (0.6)	16 (1, 17)		28 (28, 32)		28 (26, 30)	
Education			0.306		0.494		0.229
Primary School	19 (4.1)	13 (2, 19)		32 (28, 36)		38 (30, 47)	
Junior High School	44 (9.5)	16 (9.5, 18.8)		33 (29.5, 36)		38 (34.3, 43.5)	
High School	62 (13.4)	16.5 (10.8, 23.3)		33 (29, 37)		40 (31, 46)	
University and above	337 (72.9)	16 (10, 22)		34 (29, 37)		41 (34, 47)	
Employment			0.043		0.663		0.020
Full-time	365 (79.0)	16 (9, 22)		34 (29, 37)		40 (32.5, 47)	
Part-time	41 (8.9)	14 (9.5,23)		33 (27, 37)		41 (35,50)	
Retired	50 (10.8)	17.5 (14, 23)		33 (30.8, 37)		41.5 (36, 45.3)	
Loss of labour force	6 (1.3)	1 (0, 17.5)		31.5 (27, 34.8)		30 (28.8, 32)	
Frequency of gout medical consultations			0.241		0.502		0.080
At least every 3 months	61 (13.2)	16 (14, 22)		33 (27, 37)		43 (38, 48)	
Every 6 months	62 (13.4)	16 (11.8,23.3)		33 (28.8, 36)		38 (33.8,45)	
Annually	69 (14.9)	17 (10.5, 22.5)		33 (30, 37)		40 (32.5, 44.5)	
Less than once a year	270 (58.4)	15 (8, 22)		34 (29, 37)		40.5 (32, 48)	
Age of Onset			0.005		0.073		0.158
18–24 years	74 (16.0)	14.5 (7.8, 20.3)		32 (27, 36.3)		36.5 (30, 49)	
25–34 years	152 (32.9)	18 (11,24)		34 (29, 38)		41 (35,47)	
35–50 years	164 (35.5)	16 (10.3, 22)		34 (31, 37)		41 (37, 46)	
51 years and above	72 (15.6)	14 (7.3, 18)		33 (29, 37)		40 (32.3, 46)	
Family History			<0.001		0.610		0.065
Yes	61 (13.2)	18 (13,25)		34 (28.5, 37)		40 (36,48.5)	
No	301 (65.2)	16 (11, 22)		34 (29, 37)		41 (34, 47)	
Unclear	100 (21.6)	13 (4, 20.8)		33 (29, 37)		38 (31, 46)	
Alcohol History			0.008		0.290		0.191
Yes	227 (49.1)	17 (12,23)		34 (29, 37)		41 (35,47)	
No	217 (47.0)	15 (7, 21)		33 (29, 37)		40 (32, 46)	
Unclear	18 (3.9)	14.5 (0, 23.3)		30.5 (27.8, 35.3)		35.5 (30, 46.3)	
Times of Gout Attacks in the Past Year			0.035		0.506		0.432
No attacks	250 (54.1)	14 (7, 22)		33.5 (29, 37)		40 (32, 48)	
1 attack	92 (19.9)	16.5 (11,22)		34 (30, 37)		40.5 (35.3,47)	
2 attacks	65 (14.1)	17 (12, 23)		33 (28.5, 37)		38 (31, 45)	
3–5 attacks	30 (6.5)	18.5 (13, 22.3)		34 (30.5, 37.3)		40 (36.8, 46.3)	
More than 5 attacks	25 (5.4)	16 (14.5, 20.5)		35 (32, 40)		42 (38.5, 46.5)	
Duration of Gout			<0.001		0.004		0.031
Less than 1 year	227 (49.1)	13 (6, 20)		33 (27, 36)		40 (31, 48)	
1–2 years	102 (22.1)	18 (13,25)		34 (30.8, 37)		40 (35,46)	
2–5 years	71 (15.4)	17 (13, 22)		33 (31, 37)		40 (34, 44)	
5–10 years	36 (7.8)	20 (14.3, 24)		36 (32, 38.8)		45 (40, 47)	
More than 10 years	26 (5.6)	16.5 (13.5, 23.3)		33.5 (27.8, 38.3)		43.5 (38, 48.3)	
Type of Gout			<0.001		0.061		<0.001
Acute gout	132 (28.6)	21 (15, 25.8)		34 (31, 37)		42 (37, 47)	
Chronic gout	58 (12.6)	18.5 (13, 23)		35 (27.8, 38.3)		44 (38,48)	
Unclear	272 (58.9)	13.5 (6, 18)		33 (29, 36)		38 (31, 46)	
Previous Medications Taken (Multiple Choices Allowed)							
Colchicine	115 (24.9)						
Nonsteroidal anti-inflammatory drugs (NSAIDs)	70 (15.2)						
Analgesics	143 (31.0)						
Corticosteroids	17 (3.7)						
Allopurinol	52 (11.3)						
Benzbromarone	67 (14.5)						
Sodium bicarbonate	48 (10.4)						
Traditional Chinese medicine	213 (46.1)						
Do you have any Gout-related Complications? (Multiple Choices Allowed)							
Hypertension	113 (24.5)						
Hyperlipidemia	126 (27.3)						
Diabetes	43 (9.3)						
Obesity	168 (36.4)						
Coronary heart disease	17 (3.7)						
Chronic kidney disease	20 (4.3)						
Hyperthyroidism	18 (3.9)						
Hypothyroidism	11 (2.4)						
Anemia	109 (23.6)						
Psoriasis	19 (4.1)						

Bold values indicate *P* < 0.05.

### Distribution analysis of the KAP scores

Most respondents (approximately 65.5%) exhibited limited knowledge of gout, particularly in aspects related to medication and disease management. About two-thirds (67.1%) correctly distinguished gout from osteoporosis (Q1), but more than half (around 58.2%) were unable to identify uric acid–lowering drugs such as allopurinol or benzbromarone (Q12) or recognize medications that may trigger gout attacks (Q10). Fewer than half (43.6%) understood the necessity of long-term urate-lowering therapy (Q14), and 52.3% were unclear about the difference between chronic and acute gout (Q3), indicating substantial gaps in treatment awareness and disease understanding.

Participants’ attitudes toward gout were mixed. Nearly half (48.9%) disagreed with the misconception that gout mainly affects older men (Q1) and recognized the importance of following physicians’ advice for long-term treatment (Q4). However, 56.4% underestimated the systemic effects of gout beyond joint pain (Q7), and 41.7 remained concerned about the potential harm of long-term medication use (Q5). Overall, while moderate awareness of lifestyle management existed, inconsistencies in perception reflected uncertainty and ambivalence toward gout control.

In terms of practice, self-management behaviors were generally suboptimal. Fewer than half (46.8%) reported regularly improving their diet and exercise habits or attending medical follow-ups, and only a minority (29.7%) maintained continuous urate-lowering therapy or sought reliable disease-related information (Q11, Q14). Although some patients practiced dietary moderation and hydration, gaps remained in consistent adherence and proactive disease monitoring. These findings suggest that while certain awareness and behaviors exist, deficiencies persist across all three KAP dimensions. Detailed item-level data are presented in Supplementary Tables S1, S2 and Supplementary Figure S1.

### Analysis of factors associated with KAP scores

Correlation analysis showed that significant positive correlations were found between knowledge and attitude (r = 0.456, *P <* 0.001), as well as knowledge and practice (r = 0.539, *P <* 0.001). Meanwhile, there were also positive correlations between attitude and practice (r = 0.477, *P <* 0.001) (Table 2).

**Table 2:** Correlation analysis.

Characteristics	Knowledge	Attitude	Practice
Knowledge	1.000	/	/
Attitude	0.456 (*P <* 0.001)	1.000	/
Practice	0.539 (*P <* 0.001)	0.477 (*P <* 0.001)	1.000

### Multivariate logistic regression analysis

Multivariate logistic regression analysis was performed using variables with *p <* 0.25 in univariate analysis as entry criteria. Results showed that being retired (OR = 3.935, 95% CI: 1.494–10.362, *p* = 0.006), having 3–5 gout attacks in the past year (OR = 0.379, 95% CI: 0.144–0.999, *p* = 0.050), gout duration of 1–2 years (OR = 1.958, 95% CI: 1.034–3.709, *p* = 0.039), 2–5 years (OR = 2.159, 95% CI: 1.006–4.633, *p* = 0.048), and 5–10 years (OR = 4.288, 95% CI: 1.582–11.622, *p* = 0.004), and being unsure of their gout type (OR = 0.326, 95% CI: 0.193–0.551, *p <* 0.001) were independently associated with knowledge scores (Supplementary Table S3). For attitude scores, being aged 25–34 years (OR = 2.376, 95% CI: 1.075–5.251, *p* = 0.032) and 35–50 years (OR = 2.490, 95% CI: 1.015–6.108, *p* = 0.046), and having a gout duration of 5–10 years (OR = 2.459, 95% CI: 1.119–5.406, *p* = 0.025) were independently associated with more positive attitudes (Supplementary Table S4). For practice scores, being unsure of their gout type (OR = 0.430, 95% CI: 0.262–0.706, *p <* 0.001) was independently associated with suboptimal practice (Supplementary Table S5). Regarding the total KAP score, being married (OR = 2.152, 95% CI: 1.029–4.501, *p* = 0.042), consulting every 6 months compared with at least every 3 months (OR = 0.412, 95% CI: 0.174–0.975, *p* = 0.044), having a gout duration of 5–10 years (OR = 5.110, 95% CI: 1.754–14.891, *p* = 0.003), and having an unclear gout type (OR = 0.251, 95% CI: 0.140–0.450, *p <* 0.001) were independently associated with overall KAP performance (Supplementary Table S6).

### Structural equation modeling

**Figure 1:**
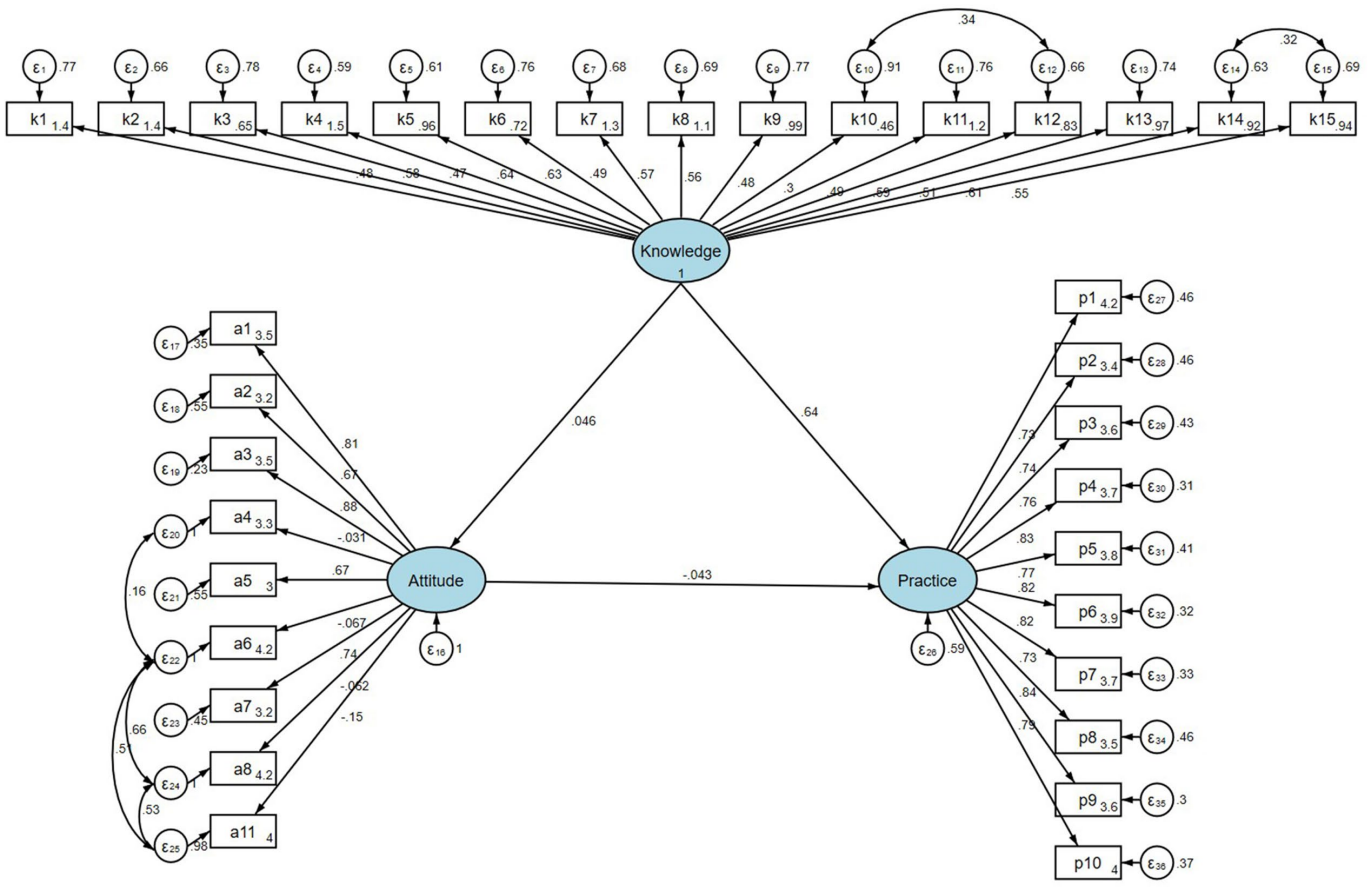
Structural equation model of factors influencing knowledge, attitude, and practice.

The fit of the SEM model yielded good indices demonstrating good model fit (RMSEA value: 0.068, SRMR value: 0.069, TLI value: 0.839, and CFI value: 0.851) (Table 3), and the results of the mediation analysis shown that knowledge had direct effect on practice (*β* = 0.644, *p <* 0.001). However, the direct effect of knowledge on attitude, the direct effect of attitude on practice, and the indirect effect of knowledge on practice were not significant (Table 4 and Figure 1).

**Table 3:** Model fitting index.

Indicators	Reference	Results
RMSEA	<0.08	0.068
SRMR	<0.08	0.069
TLI	>0.80	0.839
CFI	>0.80	0.851

**Table 4:** The direct and indirect effects among the variables.

Model paths	Total effects		Direct effect		Indirect effect	
	*β* (95%CI)	*P*	*β* (95%CI)	*P*	*β* (95%CI)	*P*
Attitude						
Knowledge	0.046 (−0.060, 1.151)	0.397	0.046 (−0.060, 1.151)	0.397		
Practice						
Knowledge	0.642 (0.577, 0.706)	<0.001	0.644 (0.579, 0.708)	<0.001	−0.002 (−0.008, 0.004)	0.537
Attitude	−0.043 (−0.126, 0.040)	0.308	−0.043 (−0.126, 0.040)	0.308		

## Discussion

Patients with gout had insufficient knowledge, negative attitude and proactive practice toward gout. Although our findings show significant positive correlations between knowledge, attitudes, and practice scores, the SEM analysis revealed that knowledge has a direct effect on practice, while the direct effects of attitudes on practice and indirect effects through attitudes were not statistically significant. The observed direct path from knowledge to practice suggests that patients’ behaviors are primarily driven by clinical guidance. This aligns with the Chinese healthcare context, where respect for medical authority often leads to high behavioral compliance regardless of personal attitudes.

This study examined knowledge, attitude, and practice scores among gout patients, revealing significant variations across different patient demographics and clinical characteristics. A previous study developed a web-based, patient-tailored tool designed to enhance adherence to urate-lowering therapy in patients with gout, which received positive evaluations (20).

These findings have crucial implications for improving gout management. Differences in knowledge, attitudes, and practices were observed across patient subgroups according to age, marital status, employment status, gout duration, and gout type, indicating the need for tailored educational approaches (21, 22). Notably, age-stratified analysis revealed significant differences across KAP domains: younger patients (18–24 years) demonstrated lower knowledge and less positive attitudes compared with middle-aged groups (25–50 years), who showed higher odds of adequate knowledge and more positive attitudes toward gout management. This may reflect greater health literacy, more frequent healthcare engagement, and longer cumulative disease experience in middle-aged patients. These findings underscore the importance of age-targeted educational interventions, particularly for younger patients who may underestimate the chronic and systemic nature of gout. Disease duration and attack frequency also emerged as important determinants of KAP. Patients with a gout duration of 1–2 years and 5–10 years demonstrated significantly higher knowledge scores compared with those diagnosed within the past year, suggesting that accumulated disease experience fosters greater understanding of gout pathophysiology and management. Similarly, patients with longer disease duration (5–10 years) exhibited more positive attitudes, likely reflecting adaptation to chronic illness and greater engagement with healthcare providers over time. In contrast, newly diagnosed patients may struggle to adjust to lifestyle and dietary modifications, highlighting the need for intensive early education at the point of diagnosis. Regarding attack frequency, the multivariate analysis showed that patients who experienced 3–5 attacks in the past year had lower odds of adequate knowledge (OR = 0.379, *p* = 0.050), which may reflect a paradox in which frequent flares are associated with disease uncertainty or inadequate management rather than improved understanding. These indicators, disease duration and attack frequency, reflect patients’ prior disease experience and cognitive engagement with their condition, and should be considered when designing individualized patient education programs. For example, younger and employed patients tended to have lower knowledge and practice scores, while longer disease duration was associated with more positive attitudes, suggesting that patient experience influences understanding and behavior (23, 24). Compared with a study in Saudi Arabia (25), where patients showed high satisfaction, adequate knowledge, and positive attitudes toward gout management, patients in Xiamen demonstrated greater knowledge gaps and more negative perceptions, particularly regarding medication adherence and dietary control. These regional contrasts highlight the influence of sociocultural and healthcare contexts on disease perception and self-management (22, 26). Notably, many participants remained unclear about the distinction between acute and chronic gout, the appropriate use of uric acid–lowering drugs, and the role of exercise in gout prevention and flare control, with 56.7% believing that intense physical activity could trigger attacks (27). Furthermore, while patients generally demonstrated awareness of dietary triggers (e.g., 80.7% correctly identified seafood as a trigger), significant gaps persisted in medication-related knowledge. Only 11.9% correctly identified febuxostat, 34.2% identified benzbromarone, and 31.2% identified allopurinol as uric acid–lowering drugs, highlighting a critical deficit in pharmacological knowledge. Low adherence to long-term urate-lowering therapy remains a critical challenge, as many patients discontinue medication during asymptomatic periods, which can worsen disease progression. These findings underscore the urgent need for evidence-based patient education and behavioral interventions that strengthen disease-specific knowledge and promote long-term adherence (28–30). Healthcare providers should prioritize patient-centered strategies that correct misconceptions about gout’s age and gender susceptibility (26, 31), emphasize the importance of sustained urate-lowering therapy, and integrate dietary and lifestyle counseling into clinical practice to enhance adherence and outcomes.

The findings on self-management practices among patients with gout provide valuable insights into how individuals actively engage in disease control. Many patients demonstrated proactive behaviors such as dietary adjustments, regular exercise, weight management, and adherence to ULT, reflecting a strong sense of responsibility and awareness toward gout management. Their efforts to monitor purine-rich foods, follow medical advice, and seek reliable information about gout and prescribed medications indicate a positive orientation toward self-care. However, important gaps remain: while dietary awareness was relatively high (e.g., 80.7% correctly identified seafood as a trigger), medication-related knowledge and adherence were notably deficient. Only 45.9% of patients were aware that chronic gout often requires lifelong urate-lowering therapy, and fewer than half correctly identified common uric acid–lowering drugs. Patients with longer disease duration (5–10 years) generally performed better in self-management, likely due to accumulated experience and repeated clinical interactions, whereas newly diagnosed patients faced greater challenges in adapting to the required lifestyle and pharmacological changes. This highlights the critical window of early disease management, where targeted education on medication adherence, particularly regarding allopurinol and other urate-lowering agents, can have the greatest impact. The SEM results further revealed that knowledge exerts a direct influence on practice, underscoring the pivotal role of patient education in improving self-management behaviors (22, 24, 32). Although attitudes toward gout also correlated positively with practice, their mediating effect was limited, suggesting that practical guidance and actionable knowledge may have a more immediate impact on behavior than attitudinal shifts. These findings highlight the importance of targeted, evidence-based educational interventions that provide clear, context-specific instructions and address common misconceptions about gout. By integrating patient-centered education with behavioral strategies—such as lifestyle counseling, peer support, and social-environmental considerations—healthcare providers can promote sustained adherence and achieve more effective, holistic management of gout.

The multivariate logistic regression analysis reveals important insights for improving clinical practice in gout management. Retirees are more likely to possess adequate gout knowledge, highlighting the need for tailored education for the non-retired population. Uncertainty about gout type is associated with suboptimal practice, emphasizing the importance of clear communication to ensure adherence to recommended actions. Surprisingly, younger age groups (25–34 years and 35–50 years) exhibit more positive attitudes toward gout management, suggesting potential effectiveness in attitude-focused interventions for these demographics. Individuals managing gout for an extended duration demonstrate a more positive attitude, indicating the significance of sustained patient engagement and support during long-term management (23, 24).

### Limitations

Limitations of this study include the use of a cross-sectional design, which restricts our ability to establish causal relationships between variables. The data collected relied on self-reported responses from participants, which may introduce recall and social desirability biases. Additionally, the study was conducted in a specific geographic region (Xiamen), which may limit the generalizability of the findings to broader populations. Furthermore, the reliance on questionnaires to assess knowledge, attitude, and practice may not capture the full complexity of patient experiences and behaviors related to gout. The relatively high educational status of participants (72.9% with university education or above) may limit the generalizability of our findings to less educated populations, who may face greater challenges in accessing and understanding gout information and implementing self-management practices. The use of an online survey platform (Questionnaire Star via WeChat) may have reduced participation among older adults or individuals without smartphones, potentially introducing a digital access bias that could affect the representativeness of the sample. However, several measures were taken to mitigate this limitation: WeChat has an extremely high penetration rate in China (over 1.3 billion monthly active users), trained researchers were present at both survey sites to assist participants with questionnaire completion, and the platform was configured to allow only one submission per WeChat account and per IP address to prevent duplicate entries. Nonetheless, this potential selection bias should be considered when interpreting the findings. Regarding data quality, a total of 160 questionnaires were excluded from the final analysis. The primary reasons for exclusion were abnormal completion times (<110 s or >1800 s; *n =* 101) and logical errors in responses such as simultaneously selecting a correct answer and “unclear” for the same item (*n =* 59), rather than system failures. Of the excluded questionnaires, 28 (17.5%) were from participants aged 51 years and older, which is within the expected range and does not suggest disproportionate exclusion of older participants. No major technical failures of the survey platform were identified during the data collection period.

## Conclusion

Patients with gout had insufficient knowledge, negative attitude and proactive practice toward gout. To optimize clinical practice in gout management, it is advisable to prioritize comprehensive patient education, particularly targeting the non-retired population. Additionally, addressing any negative attitudes observed among younger individuals and enhancing communication concerning gout types and treatment recommendations are essential steps for improving overall patient care in gout management.

## Data Availability

The original contributions presented in the study are included in the article/Supplementary material, further inquiries can be directed to the corresponding author.

## Footnotes

1
https://www.msdmanuals.cn/professional/SearchResults?query=%E7%979B%E9%A3%8E
