# Supplementary material for: Knowledge, attitudes, and practices regarding gout management among patients in Xiamen, China: a cross-sectional study
Source: Front Public Health. 2026 Jun 17;14:1786934. doi: 10.3389/fpubh.2026.1786934 (PMC13318878; doi:10.3389/fpubh.2026.1786934)
Supplement: Supplementary file 1 [file Supplementary_file_1.docx]

**Questionnaire**

**Cognition, attitude and practice of gout patients in Xiamen area**

**Part 1: Basic Patient Information**

| **Gender** |
| --- |
| Male |
| Female |
| **Age (years)** |
| 18-24 |
| 25~34 |
| 35~50 |
| 51 and above |
| **Marital Status** |
| Unmarried |
| Married |
| Divorced |
| Widowed |
| **Education** |
| Primary School |
| Junior High School |
| High School |
| University & above |
| **Employment** |
| Full-time |
| Part-time |
| Retired |
| Loss of labour force |
| **Frequency of gout medical consultations** |
| At least every 3 months |
| Every 6 months |
| Annually |
| Less than once a year |
| **Age of Onset** |
| 18-24 years |
| 25~34 years |
| 35~50 years |
| 51 years and above |
| **Family History** |
| Yes |
| No |
| Unclear |
| **Alcohol History** |
| Yes |
| No |
| Unclear |
| **Times of Gout Attacks in the Past Year** |
| No attacks |
| 1 attack |
| 2 attacks |
| 3-5 attacks |
| More than 5 attacks |
| **Duration of Gout** |
| Less than 1 year |
| 1-2 years |
| 2-5 years |
| 5-10 years |
| More than 10 years |
| **Type of Gout** |
| Acute gout |
| Chronic gout |
| Unclear |
| **Previous Medications Taken (Multiple Choices Allowed)** |
| Colchicine |
| Nonsteroidal anti-inflammatory drugs (NSAIDs) |
| Analgesics |
| Corticosteroids |
| Allopurinol |
| Benzbromarone |
| Sodium bicarbonate |
| Traditional Chinese medicine |
| **Do you have any Gout-related Complications? (Multiple Choices Allowed)** |
| Hypertension |
| Hyperlipidemia |
| Diabetes |
| Obesity |
| Coronary heart disease |
| Chronic kidney disease |
| Hyperthyroidism |
| Hypothyroidism |
| Anemia |
| Psoriasis |

**Part II: Patients' Perceptions of Gout**

**Is gout the same as osteoporosis?**

a. Not the same b. The same c.Unclear

**What are the causes of gout? (Multiple choices allowed)**

a. Calcium deficiency b. Excess uric acid c. Purine metabolism disorder

d. Kidney disease e.Unclear

**Are you aware of the difference between chronic gout and acute gout?**

a.Yes b.No c.Unclear

**How to determine if you are experiencing an acute gout attack?**

a. Severe joint pain b. Joint swelling c. Skin itching

d. Difficulty in movement e.Unclear

**Congenital enzyme deficiency can cause hereditary gout. Is gout likely to be** **hereditary?**

a.Yes b.No c.Unclear

**Gout is associated with which of the following diseases? (Multiple choices allowed)**

a. Anemia b. Hypertension c. Diabetes

d. Elevated blood lipids e.Unclear

**Which foods may trigger gout? (Multiple choices allowed)**

a. Seafood b. Sweet potatoes c. Beef d. Beer e.Unclear

**Which foods are suitable for gout patients? (Multiple choices allowed)**

a. Eggs, milk b. Cucumber, apple c. Fish soup, organ meats

d. Celery, cabbage e.Unclear

**Which of the following conditions can lead to gout attacks? (Multiple choices allowed)**

a. Cold weather b. Intense exercise c. Abstinence from alcohol d. Lack of sleep e.Unclear

**Which of the following medications pose a risk of gout? (Multiple choices allowed)**

a. Eprosartan b. Cyclosporine c. Tacrolimus

d. Levodopa Unclear

**Which lifestyle habits can improve gout? (Multiple choices allowed)**

a. Drinking 2000ml of water daily bExercising for 2 hours daily

c. Drinking wine before bedtime d. Maintaining a healthy weight

e.Unclear

**Which of the following medications are uric acid-lowering drugs? (Multiple choices allowed)**

a. Febuxostat b. Benzbromarone c. Febuxostat

d. Allopurinol Unclear

**When gout suddenly strikes, which treatment method is most effective?**

a. Immediate exercise b. Allopurinol

c. Nonsteroidal anti-inflammatory drugs like ibuprofen, aspirin, and diclofenac

d. Benzbromarone e.Unclear

**Do you know that patients with chronic gout often need to take uric acid-lowering medications for a long time, even for life?**

a. Yes b. No c. Never heard of

**Chronic gouty arthritis is a chronic condition. If the medication is stopped halfway, will it worsen the patient's condition?**

a.Yes b.No c.Unclear

**Part III: Patient attitudes towards gout**

**Do you think gout is mainly a disease of the elderly, and young people rarely suffer from gout?**

a.Strongly Agree b. Agree c.Neutral

d. Disagree e. Strongly Disagree

**Do you believe that gout is primarily a male disease, and females rarely have gout?**

a.Strongly Agree b. Agree c.Neutral

d. Disagree e. Strongly Disagree

**Do you think that chronic gout patients only need treatment when they have a gout attack, and they don't need medication at other times?**

a.Strongly Agree b. Agree c.Neutral

d. Disagree e. Strongly Disagree

**If a doctor advises you to take medication long-term to control the development of gout, would you follow the doctor's advice?**

a. I will definitely follow the advice and take medication long-term

b. I may follow the advice and take medication long-term

c. I need to carefully consider it

d. I am not very likely to follow the advice and don't want to take medication long-term

e. I do not follow the advice and refuse to take medication long-term

**Do you believe that taking too much gout medication is harmful to the body, and even if gout flares up, you will not take medication and wait for it to resolve on its own?**

a.Strongly Agree b. Agree c.Neutral

d. Disagree e. Strongly Disagree

**Do you think that dietary habits are crucial for managing gout?**

a.Strongly Agree b. Agree c.Neutral

d. Disagree e. Strongly Disagree

**Do you believe that gout only causes joint pain and does not affect other parts of the body?**

a.Strongly Agree b. Agree c.Neutral

d. Disagree e. Strongly Disagree

**If you have gout, are you worried about other complications?**

a.Strongly Agree b. Agree c.Neutral

d. Disagree e. Strongly Disagree

**Do you think gout affects your daily life or work?**

a. Significantly affected b. Occasionally affected c.Unclear

d. Not greatly affected e. Completely unaffected

**Do you believe that gout affects your emotions?**

a. Significantly affected b. Occasionally affected c.Unclear

d. Not greatly affected e. Completely unaffected

**Are you optimistic about gout and have confidence in its treatment?**

a. Very confident b. Confident c.Neutral

d. Not very confident e. Completely lacking confidence

**Part 4: Patients' Practical Actions on Improving Gout**

**Do you often improve your diet and exercise habits while reasonably losing weight?**

a. Always b. Often c.Unclear d.Rarely e.Never

**For chronic gout, would you persistently take urate-lowering therapy (ULT) medications?**

a. Always b. Often c.Unclear d.Rarely e.Never

**To control gout, would you carefully check whether specific foods are high-purine foods?**

a. Always b. Often c.Unclear d.Rarely e.Never

**Would you follow medical advice and have regular check-ups for gout-related parameters?**

a. Always b. Often c.Unclear d.Rarely e.Never

**Will you self-educate on gout-related knowledge through reliable sources and avoid relying on folk remedies or secrets?**

a. Always b. Often c.Unclear d.Rarely e.Never

**Will you actively monitor and check for complications of gout in yourself?**

a. Always b. Often c.Unclear d.Rarely e.Never

**During gatherings or social events, will you adhere to gout dietary guidelines and request understanding and support from friends, colleagues, clients, etc.?**

a. Always b. Often c.Unclear d.Rarely e.Never

**Will you communicate your psychological feelings and self-management experiences with fellow gout patients?**

a. Always b. Often c.Unclear d.Rarely e.Never

**Will you actively seek information about the names, dosages, precautions, and adverse reactions of the gout medications you are taking?**

a. Always b. Often c.Unclear d.Rarely e.Never

**To improve and treat gout, will you maintain a regular diet and quit smoking and drinking?**

a. Always b. Often c.Unclear d.Rarely e.Never

Table S1: Knowledge Dimension Score Distribution

|  | a. Not the same (1) | | b. The same (0) | | | c.Unclear(0) | |
| --- | --- | --- | --- | --- | --- | --- | --- |
| 1. Is gout the same as osteoporosis? | **308(66.7)** | | 24(5.2) | | | 130(28.1) | |
|  | a. Calcium deficiency (1) | b. Excess uric acid (1) | | c. Purine metabolism disorder (1) | d. Kidney disease (1) | | e.Unclear(0) |
| 1. What are the causes of gout? (Multiple choices allowed) | 53(11.5) | **319(69.0)** | | 260(56.3) | 101(21.9) | | 82(17.7) |
|  | a.Yes(1) | | b.No(0) | | | c.Unclear(0) | |
| 1. Are you aware of the difference between chronic gout and acute gout? | 138(29.9) | | **187(40.5)** | | | 137(29.7) | |
|  | a. Severe joint pain (1) | b. Joint swelling (1) | | c. Skin itching (0) | d. Difficulty in movement (1) | | e.Unclear(0) |
| 1. How to determine if you are experiencing an acute gout attack? | **227(49.1)** | 72(15.6) | | 12(2.6) | 22(4.8) | | 129(27.9) |
|  | a. Yes(1) | | b. No(0) | | | c.Unclear(0) | |
| 1. Congenital enzyme deficiency can cause hereditary gout. Is gout likely to be hereditary? | **222(48.1)** | | 35(7.6) | | | 205(44.4) | |
|  | a. Anemia (0) | b. Hypertension (1) | | c. Diabetes (1) | d. Elevated blood lipids (1) | | e.Unclear(0) |
| 1. Gout is associated with which of the following diseases? (Multiple choices allowed) | 57(12.3) | 153(33.1) | | 158(34.2) | **216(46.8)** | | 167(36.1) |
|  | a. Seafood (1) | b. Sweet potatoes (0) | | c. Beef (1) | d. Beer (1) | | e.Unclear(0) |
| 1. Which foods may trigger gout? (Multiple choices allowed) | **373(80.7)** | 22(4.8) | | 89(19.3) | 352(76.2) | | 64(13.9) |
|  | a. Eggs, milk (1) | b. Cucumber, apple (1) | | c. Fish soup, organ meats (0) | d. Celery, cabbage (1) | | e.Unclear(0) |
| 1. Which foods are suitable for gout patients? (Multiple choices allowed) | 231(50.0) | **287(62.1)** | | 47(10.2) | 274(59.3) | | 81(17.5) |
|  | a. Cold weather (1) | b. Intense exercise (1) | | c. Abstinence from alcohol (0) | d. Lack of sleep (1) | | e.Unclear(0) |
| 1. Which of the following conditions can lead to gout attacks? (Multiple choices allowed) | 222(48.1) | **262(56.7)** | | 39(8.4) | 206(44.6) | | 103(22.3) |
|  | a. Eprosartan (1) | b. Cyclosporine (1) | | c. Tacrolimus (1) | d. Levodopa (1) | | Unclear(0) |
| 1. Which of the following medications pose a risk of gout? (Multiple choices allowed) | 51(11.0) | 61(13.2) | | 55(11.9) | 39(8.4) | | **351(76.0)** |
|  | a. Drinking 2000ml of water daily (1) | bExercising for 2 hours daily (1) | | c. Drinking wine before bedtime (0) | d. Maintaining a healthy weight (1) | | e.Unclear(0) |
| 1. Which lifestyle habits can improve gout? (Multiple choices allowed) | **329(71.2)** | 259(56.1) | | 20(4.3) | 248(53.7) | | 72(15.6) |
|  | a. Febuxostat (1) | b. Benzbromarone (1) | | c. Febuxostat (1) | d. Allopurinol (1) | | Unclear(0) |
| 1. Which of the following medications are uric acid-lowering drugs? (Multiple choices allowed) | 55(11.9) | 158(34.2) | | 150(32.5) | 144(31.2) | | **205(44.4)** |
|  | a. Immediate exercise (0) | b. Allopurinol (0) | | c. Nonsteroidal anti-inflammatory drugs like ibuprofen, aspirin, and diclofenac (1) | d. Benzbromarone (0) | | e.Unclear(0) |
| 1. When gout suddenly strikes, which treatment method is most effective? | 8(1.7) | 32(6.9) | | **225(48.7)** | 27(5.8) | | 170(36.8) |
|  | a. Yes(1) | | b. No(0) | | | c. Never heard of(0) | |
| 1. Do you know that patients with chronic gout often need to take uric acid-lowering medications for a long time, even for life? | **212(45.9)** | | 131(28.4) | | | 119(25.8) | |
|  | a.Yes(1) | | b.No(0) | | | c.Unclear(0) | |
| 1. Chronic gouty arthritis is a chronic condition. If the medication is stopped halfway, will it worsen the patient's condition? | **216(46.8)** | | 40(8.7) | | | 206(44.6) | |

## Table S2: Attitude Dimension Score Distribution

|  | a.Strongly Agree(1) | b. Agree(2) | c.Neutral(3) | d. Disagree(4) | e. Strongly Disagree(5) |
| --- | --- | --- | --- | --- | --- |
| 1. Do you think gout is mainly a disease of the elderly, and young people rarely suffer from gout? | 30(6.5) | 35(7.6) | 86(18.6) | **233(50.4)** | 78(16.9) |
|  | a.Strongly Agree(1) | b. Agree(2) | c.Neutral(3) | d. Disagree(4) | e. Strongly Disagree(5) |
| 1. Do you believe that gout is primarily a male disease, and females rarely have gout? | 31(6.7) | 57(12.3) | 105(22.7) | **215(46.5)** | 54(11.7) |
|  | a.Strongly Agree(1) | b. Agree(2) | c.Neutral(3) | d. Disagree(4) | e. Strongly Disagree(5) |
| 1. Do you think that chronic gout patients only need treatment when they have a gout attack, and they don't need medication at other times? | 25(5.4) | 37(8.0) | 99(21.4) | **221(47.8)** | 80(17.3) |
|  | a. I will definitely follow the advice and take medication long-term (5) | b. I may follow the advice and take medication long-term (4) | c. I need to carefully consider it (3) | d. I am not very likely to follow the advice and don't want to take medication long-term (2) | e. I do not follow the advice and refuse to take medication long-term (1) |
| 1. If a doctor advises you to take medication long-term to control the development of gout, would you follow the doctor's advice? | **191(41.3)** | 123(26.6) | 81(17.5) | 48(10.4) | 19(4.1) |
|  | a.Strongly Agree(1) | b. Agree(2) | c.Neutral(3) | d. Disagree(4) | e. Strongly Disagree(5) |
| 1. Do you believe that taking too much gout medication is harmful to the body, and even if gout flares up, you will not take medication and wait for it to resolve on its own? | 42(9.1) | 56(12.1) | 127(27.5) | **177(38.3)** | 60(13.0) |
|  | a.Strongly Agree(5) | b. Agree(4) | c.Neutral(3) | d. Disagree(2) | e. Strongly Disagree(1) |
| 1. Do you think that dietary habits are crucial for managing gout? | 168(36.4) | **183(39.6)** | 75(16.2) | 19(4.1) | 17(3.7) |
|  | a.Strongly Agree(1) | b. Agree(2) | c.Neutral(3) | d. Disagree(4) | e. Strongly Disagree(5) |
| 1. Do you believe that gout only causes joint pain and does not affect other parts of the body? | 42(9.1) | 36(7.8) | 94(20.3) | **224(48.5)** | 66(14.3) |
|  | a. Strongly concerned (5) | b. Concerned (4) | c.Neutral(3) | d. Not concerned (2) | e. Completely unconcerned (1) |
| 1. If you have gout, are you worried about other complications? | 140(30.3) | **200(43.3)** | 90(19.5) | 20(4.3) | 12(2.6) |
|  | a. Significantly affected | b. Occasionally affected | c.Unclear | d. Not greatly affected | e. Completely unaffected |
| 1. Do you think gout affects your daily life or work? | **215(46.5)** | 151(32.7) | 65(14.1) | 19(4.1) | 12(2.6) |
|  | a. Significantly affected | b. Occasionally affected | c.Unclear | d. Not greatly affected | e. Completely unaffected |
| 1. Do you believe that gout affects your emotions? | **213(46.1)** | 152(32.9) | 66(14.3) | 12(2.6) | 19(4.1) |
|  | a. Very confident (5) | b. Confident (4) | c.Neutral(3) | d. Not very confident (2) | e. Completely lacking confidence (1) |
| 1. Are you optimistic about gout and have confidence in its treatment? | 136(29.4) | **181(39.2)** | 108(23.4) | 26(5.6) | 11(2.4) |

Table S3: Univariate and multivariate statistical analysis for the Knowledge Dimension.

| **Cut-off Value: ≥16 / <16** | **No.** | **Univariate** | | **Multivariate (Regression Method = Input)** | |
| --- | --- | --- | --- | --- | --- |
|  |  | **OR(95%CI)** | **P** | **OR(95%CI)** | **P** |
| **Gender** |  |  |  |  |  |
| Male | 128/251 | ref. |  |  |  |
| Female | 109/211 | 1.027(0.712, 1.481) | 0.887 |  |  |
| **Age (years) (Comparison=Difference)** |  |  |  |  |  |
| 18-24 | 20/59 | ref. |  | ref. |  |
| 25~34 | 73/136 | 2.260(1.197, 4.267) | **0.012** | 1.648(0.672, 4.043) | 0.275 |
| 35~50 | 96/181 | 1.465(0.952, 2.256) | 0.083 | 1.174(0.402, 3.429) | 0.769 |
| 51 and above | 48/86 | 1.443(0.888,2.344) | 0.139 | 0.784(0.218,2.816) | 0.709 |
| **Marital Status** |  |  |  |  |  |
| Unmarried | 45/109 | ref. |  | ref. |  |
| Married | 190/345 | 1.743(1.127, 2.697) | **0.013** | 1.027(0.504, 2.093) | 0.941 |
| Divorced or Widowed | 2/8 | 0.474(0.091, 2.456) | 0.374 | 0.165(0.024, 1.149) | 0.069 |
| **Education (Comparison=Difference)** |  |  |  |  |  |
| Junior High School & below | 30/63 | ref. |  |  |  |
| High School | 34/62 | 0.862(0.503,1.477) | 0.588 |  |  |
| University & above | 173/337 | 1.151(0.668, 1.983) | 0.612 |  |  |
| **Employment** |  |  |  |  |  |
| Full-time | 186/365 | ref. |  | ref. |  |
| Part-time | 17/41 | 0.682(0.354, 1.311) | 0.251 | 0.758(0.355, 1.620) | 0.475 |
| Retired | 32/50 | 1.711(0.927, 3.158) | 0.086 | 3.740(1.406, 9.943) | **0.008** |
| Loss of labour force | 2/6 | 0.481(0.087, 2.660) | 0.402 | 1.349(0.218, 8.335) | 0.747 |
| **Frequency of gout medical consultations** |  |  |  |  |  |
| At least every 3 months | 34/61 | ref. |  |  |  |
| Every 6 months | 32/62 | 0.847(0.417, 1.722) | 0.647 |  |  |
| Annually | 40/69 | 1.095(0.546, 2.196) | 0.797 |  |  |
| Less than once a year | 131/270 | 0.748(0.428, 1.309) | 0.309 |  |  |
| **Age of Onset (Comparison=Difference)** |  |  |  |  |  |
| 18-24 years | 30/74 | ref. |  | ref. |  |
| 25~34 years | 90/152 | 0.955(0.494, 1.846) | 0.890 | 1.278(0.612, 2.667) | 0.514 |
| 35~50 years | 87/164 | 2.032(1.150, 3.591) | **0.015** | 1.061(0.463, 2.430) | 0.888 |
| 51 years and above | 30/72 | 1.582(0.904, 2.769) | 0.108 | 0.654(0.267, 1.602) | 0.353 |
| **Family History** |  |  |  |  |  |
| Yes | 37/61 | ref. |  | ref. |  |
| No | 161/301 | 0.746(0.425, 1.308) | 0.306 | 0.822(0.440, 1.536) | 0.539 |
| Unclear | 39/100 | 0.415(0.216, 0.796) | **0.008** | 0.500(0.238, 1.052) | 0.068 |
| **Alcohol History** |  |  |  |  |  |
| Yes | 129/227 | ref. |  | ref |  |
| No | 100/217 | 0.649(0.447, 0.944) | **0.024** | 0.985(0.635-1.529) | 0.946 |
| Unclear | 8/18 | 0.608(0.231, 1.597) | 0.312 | 1.447(0.472-4.442) | 0.518 |
| **Times of Gout Attacks in the Past Year (Comparison=Difference)** |  |  |  |  |  |
| No attacks | 114/250 | ref. |  | ref. |  |
| 1 attack | 52/92 | 1.551(0.958, 2.510) | 0.074 | 0.645(0.338, 1.232) | 0.184 |
| 2 attacks | 39/65 | 1.437(0.828, 2.494) | 0.198 | 0.611(0.286, 1.302) | 0.201 |
| 3-5 attacks | 17/30 | 1.110(0.520,2.369) | 0.787 | 0.383(0.145,1.013) | **0.053** |
| More than 5 attacks | 15/25 | 1.241(0.536, 2.869) | 0.614 | 0.561(0.195, 1.611) | 0.283 |
| **Duration of Gout (Comparison=Difference)** |  |  |  |  |  |
| Less than 1 year | 91/227 | ref. |  | ref. |  |
| 1-2 years | 62/102 | 2.316(1.436, 3.736) | **<0.001** | 1.992(1.049, 3.784) | **0.035** |
| 2-5 years | 43/71 | 1.508(0.885, 2.569) | 0.131 | 2.206(1.024, 4.750) | **0.043** |
| 5-10 years | 26/36 | 2.226(1.038, 4.776) | **0.040** | 4.327(1.592, 11.763) | **0.004** |
| More than 10 years | 15/26 | 0.956(0.422, 2.163) | 0.914 | 1.848(0.674, 5.065) | 0.233 |
| **Type of Gout** |  |  |  |  |  |
| Acute gout | 94/132 | ref. |  | ref. |  |
| Chronic gout | 37/58 | 0.712(0.370,1.371) | 0.310 | 0.610(0.302,1.232) | 0.168 |
| Unclear | 106/272 | 0.258(0.165, 0.404) | **<0.001** | 0.327(0.191, 0.561) | **<0.001** |

Multivariate analysis was performed using a logistic regression model, adjusting for variables with P < 0.25 in univariate analysis.Table S4. Univariate and multivariate statistical analysis for the Attitude Dimension

| **Cut-off Value：≥34 /**<**34** | **No.** | **Univariate** | | **Multivariate (Regression Method = Input)** | |
| --- | --- | --- | --- | --- | --- |
|  |  | **OR(95%CI)** | **P** | **OR(95%CI)** | **P** |
| **Gender** |  |  |  |  |  |
| Male | 124/251 | ref. |  |  |  |
| Female | 110/211 | 1.115(0.773, 1.609) | 0.559 |  |  |
| **Age (years) (Comparison=Difference)** |  |  |  |  |  |
| 18-24 | 17/59 | ref. |  | ref. |  |
| 25~34 | 73/136 | 2.863(1.485, 5.520) | **0.002** | 2.528(1.048, 6.101) | **0.039** |
| 35~50 | 105/181 | 3.413(1.807, 6.448) | **<0.001** | 2.671(0.944, 7.559) | **0.064** |
| 51 and above | 39/86 | 2.050(1.012, 4.151) | **0.046** | 1.356(0.397, 4.630) | 0.627 |
| **Marital Status** |  |  |  |  |  |
| Unmarried | 45/109 | ref. |  | ref. |  |
| Married | 190/345 | 1.979(1.274, 3.073) | **0.002** | 1.370(0.694, 2.708) | 0.365 |
| Divorced or Widowed | 2/8 | 0.228(0.027, 1.919) | 0.174 | 0.181(0.018, 1.783) | 0.143 |
| **Education (Comparison=Difference)** |  |  |  |  |  |
| Junior High School & below | 27/63 | ref. |  | ref |  |
| High School | 30/62 | 1.250(0.618, 2.530) | 0.535 | 1.266(0.582-2.750) | 0.552 |
| University & above | 177/337 | 1.475(0.857, 2.538) | 0.161 | 1.773(0.896-3.509) | 0.100 |
| **Employment** |  |  |  |  |  |
| Full-time | 190/365 | ref. |  | ref |  |
| Part-time | 19/41 | 0.795(0.416, 1.520) | 0.488 | 1.364(0.646-2.883) | 0.416 |
| Retired | 24/50 | 0.850(0.471, 1.536) | 0.591 | 1.525(0.630-3.693) | 0.349 |
| Loss of labour force | 1/6 | 0.184(0.021, 1.592) | 0.124 | 0.429(0.043-4.311) | 0.472 |
| **Frequency of gout medical consultations** |  |  |  |  |  |
| At least every 3 months | 30/61 | ref. |  |  |  |
| Every 6 months | 29/62 | 0.908(0.447, 1.843) | 0.789 |  |  |
| Annually | 33/69 | 0.947(0.475, 1.887) | 0.877 |  |  |
| Less than once a year | 142/270 | 1.146(0.657, 1.999) | 0.630 |  |  |
| **Age of Onset (Comparison=Difference)** |  |  |  |  |  |
| 18-24 years | 29/74 | ref. |  | ref |  |
| 25~34 years | 86/152 | 2.022(1.148, 3.562) | **0.015** | 0.909(0.445-1.860) | 0.795 |
| 35~50 years | 86/164 | 1.711(0.979, 2.990) | 0.059 | 0.911(0.412-2.015) | 0.819 |
| 51 years and above | 33/72 | 1.313(0.680, 2.535) | 0.417 | 1.128(0.484-2.625) | 0.781 |
| **Family History** |  |  |  |  |  |
| Yes | 32/61 | ref. |  |  |  |
| No | 157/301 | 0.988(0.569, 1.714) | 0.966 |  |  |
| Unclear | 45/100 | 0.741(0.392, 1.404) | 0.359 |  |  |
| **Alcohol History** |  |  |  |  |  |
| Yes | 121/227 | ref. |  | ref |  |
| No | 107/217 | 0.852(0.587, 1.237) | 0.400 | 1.117(0.733-1.702) | 0.607 |
| Unclear | 6/18 | 0.438(0.159, 1.208) | 0.111 | 0.696(0.225-2.153) | 0.530 |
| **Times of Gout Attacks in the Past Year (Comparison=Difference)** |  |  |  |  |  |
| No attacks | 125/250 | ref. |  |  |  |
| 1 attack | 47/92 | 1.044(0.648,1.685) | 0.859 |  |  |
| 2 attacks | 31/65 | 0.892(0.519, 1.534) | 0.680 |  |  |
| 3-5 attacks | 17/30 | 1.329(0.623, 2.834) | 0.461 |  |  |
| More than 5 attacks | 14/25 | 1.205(0.526, 2.757) | 0.659 |  |  |
| **Duration of Gout (Comparison=Difference)** |  |  |  |  |  |
| Less than 1 year | 103/227 | ref. |  | ref. |  |
| 1-2 years | 59/102 | 1.652(1.030,2.648) | **0.037** | 1.355(0.797,2.302) | 0.262 |
| 2-5 years | 35/71 | 0.911(0.541, 1.534) | 0.725 | 1.118(0.597, 2.092) | 0.728 |
| 5-10 years | 24/36 | 1.933(0.934, 4.000) | 0.076 | 2.618(1.092, 6.278) | **0.031** |
| More than 10 years | 13/26 | 0.820(0.366, 1.834) | 0.628 | 1.044(0.432, 2.524) | 0.924 |
| **Type of Gout** |  |  |  |  |  |
| Acute gout | 75/132 | ref. |  | ref |  |
| Chronic gout | 34/58 | 1.077(0.576, 2.013) | 0.817 | 1.005(0.510-1.979) | 0.989 |
| Unclear | 125/272 | 0.646(0.425, 0.982) | **0.041** | 0.770(0.467-1.272) | 0.308 |

Multivariate analysis was performed using a logistic regression model, adjusting for variables with P < 0.25 in univariate analysis.

Table S5. Univariate and multivariate statistical analysis for the Practice Dimension

| **Cut-off Value：≥40/**<**40** | **No.** | **Univariate** | | **Multivariate (Regression Method = Input)** | |
| --- | --- | --- | --- | --- | --- |
|  |  | **OR(95%CI)** | **P** | **OR(95%CI)** | **P** |
| **Gender** |  |  |  |  |  |
| Male | 142/251 | ref. |  |  |  |
| Female | 112/211 | 0.868(0.601, 1.255) | 0.452 |  |  |
| **Age (years) (Comparison=Difference)** |  |  |  |  |  |
| 18-24 | 23/59 | ref. |  | ref. |  |
| 25~34 | 68/136 | 0.418(0.212, 0.823) | **0.012** | 0.872(0.365, 2.081) | 0.758 |
| 35~50 | 111/181 | 0.654(0.378, 1.131) | 0.128 | 1.218(0.427, 3.472) | 0.712 |
| 51 and above | 52/86 | 1.037(0.613, 1.754) | 0.893 | 1.897(0.580, 6.201) | 0.290 |
| **Marital Status** |  |  |  |  |  |
| Unmarried | 47/109 | ref. |  | ref. |  |
| Married | 205/345 | 1.932(1.249, 2.986) | **0.003** | 1.411(0.692, 2.876) | 0.344 |
| Divorced or Widowed | 2/8 | 0.440(0.085, 2.277) | 0.327 | 0.494(0.068, 3.577) | 0.485 |
| **Education (Comparison=Difference)** |  |  |  |  |  |
| Junior High School & below | 26/63 | ref. |  | ref |  |
| High School | 34/62 | 1.728(0.851, 3.509) | 0.130 | 2.399(1.037-5.547) | 0.041 |
| University & above | 194/337 | 1.931(1.118, 3.333) | **0.018** | 4.463(2.104-9.469) | <0.001 |
| **Employment** |  |  |  |  |  |
| Full-time | 201/365 | ref. |  |  |  |
| Part-time | 24/41 | 1.152(0.599, 2.217) | 0.672 |  |  |
| Retired | 29/50 | 1.127(0.619, 2.050) | 0.696 |  |  |
| Loss of labour force | 0/6 | 0(0, 0) | 0.999 |  |  |
| **Frequency of gout medical consultations** |  |  |  |  |  |
| At least every 3 months | 41/61 | ref. |  | ref |  |
| Every 6 months | 29/62 | 0.429(0.206, 0.891) | **0.023** | 0.310(0.136-0.707) | 0.005 |
| Annually | 37/69 | 0.564(0.276, 1.152) | 0.116 | 0.478(0.210-1.088) | 0.078 |
| Less than once a year | 147/270 | 0.583(0.325, 1.047) | 0.071 | 0.732(0.362-1.479) | 0.384 |
| **Age of Onset (Comparison=Difference)** |  |  |  |  |  |
| 18-24 years | 29/74 | ref. |  | ref |  |
| 25~34 years | 90/152 | 0.610(0.316, 1.176) | 0.140 | 1.750(0.841-3.639) | 0.134 |
| 35~50 years | 98/164 | 1.373(0.781, 2.414) | 0.271 | 1.472(0.651-3.326) | 0.353 |
| 51 years and above | 37/72 | 1.405(0.804, 2.453) | 0.232 | 1.387(0.587-3.279) | 0.456 |
| **Family History** |  |  |  |  |  |
| Yes | 34/61 | ref. |  | ref |  |
| No | 178/301 | 1.149(0.660, 2.002) | 0.623 | 1.359(0.722-2.557) | 0.342 |
| Unclear | 42/100 | 0.575(0.302, 1.093) | 0.091 | 0.717(0.342-1.502) | 0.377 |
| **Alcohol History** |  |  |  |  |  |
| Yes | 131/227 | ref. |  | ref |  |
| No | 116/217 | 0.842(0.579, 1.224) | 0.367 | 1.212(0.778-1.890) | 0.395 |
| Unclear | 7/18 | 0.466(0.174, 1.247) | 0.128 | 0.833(0.266-2.612) | 0.754 |
| **Times of Gout Attacks in the Past Year (Comparison=Difference)** |  |  |  |  |  |
| No attacks | 135/250 | ref. |  |  |  |
| 1 attack | 54/92 | 1.211(0.746, 1.964) | 0.439 |  |  |
| 2 attacks | 32/65 | 0.751(0.436, 1.292) | 0.301 |  |  |
| 3-5 attacks | 17/30 | 1.114(0.522, 2.376) | 0.780 |  |  |
| More than 5 attacks | 16/25 | 1.474(0.628, 3.462) | 0.373 |  |  |
| **Duration of Gout (Comparison=Difference)** |  |  |  |  |  |
| Less than 1 year | 115/227 | ref. |  | ref. |  |
| 1-2 years | 57/102 | 1.234(0.771, 1.973) | 0.381 | 0.925(0.517, 1.655) | 0.793 |
| 2-5 years | 37/71 | 1.060(0.622, 1.807) | 0.831 | 0.881(0.429, 1.810) | 0.730 |
| 5-10 years | 28/36 | 3.409(1.490, 7.799) | **0.004** | 2.272(0.979, 7.598) | 0.055 |
| More than 10 years | 17/26 | 1.840(0.787, 4.299) | 0.159 | 1.463(0.549, 3.895) | 0.446 |
| **Type of Gout** |  |  |  |  |  |
| Acute gout | 88/132 | ref. |  | ref. |  |
| Chronic gout | 42/58 | 1.312(0.665, 2.591) | 0.433 | 1.221(0.574, 2.596) | 0.604 |
| Unclear | 124/272 | 0.419(0.272, 0.646) | **<0.001** | 0.389(0.223, 0.680) | **<0.001** |

Multivariate analysis was performed using a logistic regression model, adjusting for variables with P < 0.25 in univariate analysis.

**Table S6. Univariate and multivariate statistical analysis for the KAP Dimension**

| **Cut-off Value：>90/≤90** | **Univariate** | | **Multivariate (Regression Method = Input)** | |
| --- | --- | --- | --- | --- |
|  | **OR(95%CI)** | **P** | **OR(95%CI)** | **P** |
| **Gender** |  |  |  |  |
| Male | ref. |  |  |  |
| Female | 0.831(0.576, 1.199) | 0.322 |  |  |
| **Age (years) (Comparison=Difference)** |  |  |  |  |
| 18-24 | ref. |  | ref. |  |
| 25~34 | 2.346(1.227, 4.485) | 0.010 | 1.298(0.525, 3.207) | 0.573 |
| 35~50 | 2.573(1.375, 4.813) | 0.003 | 1.043(0.352, 3.092) | 0.939 |
| 51 and above | 2.075(1.033, 4.168) | 0.040 | 0.961(0.283, 3.259) | 0.961 |
| **Marital Status** |  |  |  |  |
| Unmarried | ref. |  | ref. |  |
| Married | 2.051(1.314, 3.201) | 0.002 | 2.024(0.966, 4.242) | 0.062 |
| Divorced or Widowed | 0.256(0.030, 2.161) | 0.211 | 0.179(0.012, 2.695) | 0.213 |
| **Education (Comparison=Difference)** |  |  |  |  |
| Junior High School & below | ref. |  |  |  |
| High School | 1.733(0.851, 3.532) | 0.130 | 2.190(0.931-5.154) | 0.073 |
| University & above | 1.615(0.931, 2.804) | 0.088 | 2.480(1.163-5.288) | 0.019 |
| **Employment** |  |  |  |  |
| Full-time | ref. |  |  |  |
| Part-time | 0.897(0.470, 1.714) | 0.743 |  |  |
| Retired | 1.126(0.623, 2.034) | 0.695 |  |  |
| Loss of labour force | - | 0.999 |  |  |
| **Frequency of gout medical consultations** |  |  |  |  |
| At least every 3 months | ref. |  |  |  |
| Every 6 months | 0.502(0.244, 1.029) | 0.060 | 0.455(0.191-1.080) | 0.074 |
| Annually | 0.687(0.344, 1.372) | 0.287 | 0.967(0.403-2.321) | 0.941 |
| Less than once a year | 0.782(0.448, 1.368) | 0.389 | 1.771(0.822-3.814) | 0.144 |
| **Age of Onset (Comparison=Difference)** |  |  |  |  |
| 18-24 years | ref. |  | ref. |  |
| 25~34 years | 2.208(1.247, 3.911) | 0.007 | 1.201(0.563, 2.562) | 0.636 |
| 35~50 years | 1.741(0.991, 3.059) | 0.054 | 1.020(0.440, 2.364) | 0.963 |
| 51 years and above | 1.243(0.639, 2.420) | 0.521 | 0.983(0.404, 2.391) | 0.969 |
| **Family History** |  |  |  |  |
| Yes | ref. |  | ref. |  |
| No | 0.889(0.512, 1.544) | 0.676 | 0.900(0.472, 1.714) | 0.748 |
| Unclear | 0.498(0.261, 0.952) | 0.035 | 0.554(0.259, 1.184) | 0.128 |
| **Alcohol History** |  |  |  |  |
| Yes | ref. |  |  |  |
| No | 0.819(0.564, 1.188) | 0.293 | 1.305(0.827-2.061) | 0.253 |
| Unclear | 0.470(0.171, 1.296) | 0.145 | 1.083(0.335-3.501) | 0.894 |
| **Times of Gout Attacks in the Past Year (Comparison=Difference)** |  |  |  |  |
| No attacks | ref. |  |  |  |
| 1 attack | 0.951(0.589, 1.535) | 0.836 | 0.491(0.245-0.986) | 0.045 |
| 2 attacks | 0.873(0.504, 1.510) | 0.626 | 0.580(0.252-1.332) | 0.199 |
| 3-5 attacks | 1.238(0.580, 2.645) | 0.581 | 0.852(0.292-2.483) | 0.769 |
| More than 5 attacks | 1.926(0.820, 4.522) | 0.132 | 1.489(0.443-4.996) | 0.520 |
| **Duration of Gout (Comparison=Difference)** |  |  |  |  |
| Less than 1 year | ref. |  | ref. |  |
| 1-2 years | 1.832(1.142, 2.937) | 0.012 | 2.064(1.056, 4.032) | 0.034 |
| 2-5 years | 0.959(0.558, 1.649) | 0.881 | 1.232(0.555, 2.733) | 0.608 |
| 5-10 years | 4.168(1.875, 9.269) | <0.001 | 5.392(1.861, 15.623) | 0.002 |
| More than 10 years | 1.895(0.833, 4.308) | 0.127 | 1.567(0.565, 4.346) | 0.388 |
| **Type of Gout** |  |  |  |  |
| Acute gout | ref. |  | ref. |  |
| Chronic gout | 0.875(0.462, 1.660) | 0.683 | 0.716(0.344, 1.487) | 0.370 |
| Unclear | 0.321(0.208, 0.495) | <0.001 | 0.229(0.127, 0.412) | <0.001 |

Figure S1: Practice Dimension Score Distribution
